# Association between quantitative CT-derived body composition and lung low-attenuation area percentage: discrimination of high LAA% burden

**DOI:** 10.3389/fmed.2026.1940602

**Published:** 2026-09-14

**Authors:** Mengyao Wang, Zhibo Ge, Xinxin Jiang, Ya-nan Zhao, Weijun Qian, Li Li

**Affiliations:** Department of Radiology, Kaifeng Central Hospital Affiliated to Henan Medical University, Kaifeng, Henan Province, China

**Keywords:** association, body composition, discrimination, low-attenuation area percentage, quantitative CT

## Abstract

**Objective:**

To investigate the associations between quantitative CT (QCT)-derived body composition parameters and CT-derived low-attenuation area percentage (LAA%), and to evaluate their ability to discriminate participants with high LAA% burden.

**Methods:**

A total of 260 individuals aged ≥40 years who underwent chest CT and QCT during health examinations at Kaifeng Central Hospital from January 2023 to January 2025 were retrospectively included. The study cohort was not defined as a clinically diagnosed COPD population, and a clinical diagnosis of COPD was not required for inclusion. Clinical and body composition data were collected, and the participants were categorized into grades 0, 1, 2, and 3 according to CT-derived LAA−950%. The general data and body composition of each group were compared. Spearman correlation analysis was used to assess unadjusted associations between QCT-derived body composition parameters and LAA%. Covariate-adjusted associations were further evaluated using Gamma generalized linear models adjusted for age, sex, and BMI. Receiver operating characteristic (ROC) curve analysis was performed to evaluate the discriminative performance of QCT-derived body composition parameters for LAA% grade 3.

**Results:**

Participants in LAA% grade 3 had the lowest pectoralis major muscle area (PMcsa), pectoralis major muscle index (PMI), pectoralis major muscle density (PMD), subcutaneous fat content, total muscle area of the same layer, and L1−2 vertebral volumetric bone mineral density (vBMD), while the total fat infiltration rate of the pectoralis major muscle was the highest (*P* < 0.05). LAA% was negatively correlated with PMcsa, PMD, PMI, subcutaneous fat content, total muscle area of the same layer, and vBMD, and positively correlated with total fat infiltration rate of pectoralis major muscle (*P* < 0.05). The apparent AUCs of the body composition parameters for discriminating LAA% grade 3 ranged from 0.733 to 0.887, while the bootstrap-corrected AUCs ranged from 0.726 to 0.881, indicating generally stable moderate-to-good discriminative performance.

**Conclusions:**

QCT-derived body composition parameters are associated with CT-derived LAA%, and several of these parameters show good discriminative performance for identifying participants with LAA% grade 3. These findings support the potential value of integrated CT-based assessment of lung attenuation and body composition.

## 1 Introduction

Chronic Obstructive Pulmonary Disease (COPD) is a common chronic respiratory disease characterized by persistent airflow limitation, one of the important pathological foundations of which is emphysema (1). Emphysema is characterized by abnormal permanent dilation and wall destruction of the distal airspaces of the terminal bronchioles, leading to a decrease in the elastic recoil force of lung tissue and gas trapping (2). High-resolution computed tomography (CT) is the gold standard for imaging assessment of emphysema, among which the emphysema index (Low Attenuation Area Percentage, LAA%) can objectively and repeatedly quantify the lesion, playing a significant role in early diagnosis, monitoring, and efficacy evaluation of the disease (3). Previous studies, particularly in COPD populations, have demonstrated associations between emphysema-related structural abnormalities and extrapulmonary changes, including skeletal muscle loss, altered fat distribution, and reduced bone mineral density (4, 5). However, CT-derived LAA% is an imaging-based measure of low-attenuation lung parenchyma and, in the absence of spirometric information, should not itself be interpreted as establishing a clinical diagnosis of COPD. Accordingly, simultaneous assessment of LAA% and CT-derived body composition may provide additional information regarding the relationship between pulmonary structural changes and systemic body composition.

Currently, the analysis of body components typically relies on multiple independent tools such as bioimpedance and dual-energy X- ray absorptiometry, which are cumbersome and costly, making it difficult to integrate them routinely into clinical practice. Most studies have demonstrated that routine chest CT itself contains rich information on chest wall and upper abdominal body components, and relevant parameters can be extracted through post-processing techniques. Previous research has indicated that quantitative computed tomography (QCT) can detect and quantify emphysema-related pulmonary structural abnormalities and assess their extent (6, 7). In this context, the CT assessment refers to emphysema-related pulmonary structural abnormalities rather than to the early detection of reduced muscle mass or bone mineral density. Although associations between emphysema-related structural abnormalities and body composition changes have been reported previously (4, 5), evaluating multiple CT-derived body composition dimensions together with quantitative LAA% within the same examination may provide complementary imaging information. Clarifying this association may improve understanding of the systemic changes associated with emphysema and may provide complementary CT-derived imaging information for characterizing individuals with a greater CT-defined emphysema burden. Based on this, this study aims to analyze the associations of QCT-derived body composition indicators, including pectoralis major muscle parameters, subcutaneous fat, total muscle area, and lumbar bone density, with LAA%, and to further evaluate their ability to discriminate participants with LAA% grade 3, thereby providing imaging evidence for the comprehensive assessment of emphysema-related structural changes.

## 2 Data and methods

### 2.1 Study subjects

We retrospectively included 260 individuals who underwent health examinations with chest CT and QCT at Kaifeng Central Hospital between January 2023 and January 2025. A clinical diagnosis of COPD was not required for inclusion, and participants were subsequently categorized according to CT-derived LAA%. The inclusion criteria were as follows: (1) age ≥40 years; (2) examination using the same 64-slice CT scanner, with imaging data available for QCT and NeuLungCARE-QA analysis; and (3) data required for the present analyses were available. The exclusion criteria were as follows: (1) other confirmed pulmonary diseases, including bronchiectasis, interstitial lung disease, active pulmonary tuberculosis, pleural effusion, pulmonary masses, pulmonary atelectasis, or previous lung resection; (2) chronic musculoskeletal diseases, myasthenia gravis, or other muscle-related diseases; (3) lumbar vertebral fracture, spinal infection, or bone tumor; (4) chest or spinal deformity; (5) tracheal diverticulum; or (6) missing data required for the present analyses. This study was approved by the Medical Research Ethics Committee of Kaifeng Central Hospital (2026ks-kt004). Because of the retrospective nature of the study, the requirement for informed consent was waived.

### 2.2 Data collection

#### 2.2.1 General data

Sex, age, height, and weight were collected from the health examination records, and body mass index (BMI) was calculated. Detailed histories of medications potentially affecting bone metabolism, including thyroid hormone therapy, postmenopausal hormone replacement therapy, systemic corticosteroids, calcium/vitamin D supplementation, and bisphosphonates, were not consistently documented in the retrospective health examination records and therefore could not be reliably included in the present analysis. Smoking history and pulmonary function test results were also not consistently available in the retrospective health examination records and therefore could not be reliably incorporated into the present analysis.

#### 2.2.2 Body composition measurement

Chest CT was performed using a Toshiba Aquilion 64-slice helical CT scanner with a Mindways QCT calibration phantom. The scanning parameters were as follows: slice thickness, 1 mm; field of view, 500 mm; tube voltage, 120 kVp; automatic tube-current modulation; matrix, 512 × 512; and standard reconstruction algorithm. The scanning range included at least the L2–L3 intervertebral disc level. QCT phantom calibration was performed before image acquisition.

The raw CT data were transferred to the QCT workstation and analyzed using Mindways QCT software. (1) Slice selection: Open the raw data on the imaging workstation, identify the upper edge of the aortic arch on the axial CT images, then scroll to the lung apex to identify the first axial image of the aortic arch as the measurement slice; (2) Pectoralis Major Muscle parameter measurement: On the measurement slice above the aortic arch, use the QCT software to outline and analyze the area of the pectoralis major muscle (Pectoralis Major Muscle Cross-Sectional Area, PMcsa) (see Figure 1). PMI was calculated as PMcsa divided by height squared and was expressed as cm^2^/m^2^, which is used to correct the effect of height on PMcsa. Additionally, the CT values are automatically measured. The average CT value of both pectoralis major muscles is calculated and then averaged to determine the pectoralis major muscle density (Pectoralis Major Muscle Density, PMD). (3) Overall pectoralis major fat infiltration rate: Using QCT processing software to outline and analyze the overall fat infiltration rate of the pectoralis major on the same plane as the aortic arch superior margin and PMcsa measurement; (4) Subcutaneous fat content measurement: Using QCT processing software to outline and measure the cross-sectional area of subcutaneous fat tissue on the same plane as the aortic arch superior margin and PMcsa measurement, as an assessment index for subcutaneous fat content; (5) Measurement of total muscle area of all muscle groups on the same layer: Using QCT processing software to outline and analyze the total muscle area of all muscle groups on the same plane as the aortic arch superior margin and PMcsa measurement (see Figure 2); (6) L_1–2_ vertebral body volume bone mineral density (Volumetric Bone Mineral Density, vBMD): Using QCT software to outline and analyze the trabecular bone mineral density perpendicular to the central levels of L1 and L2 vertebrae and take the average value (see Figure 3).

**Figure 1:**
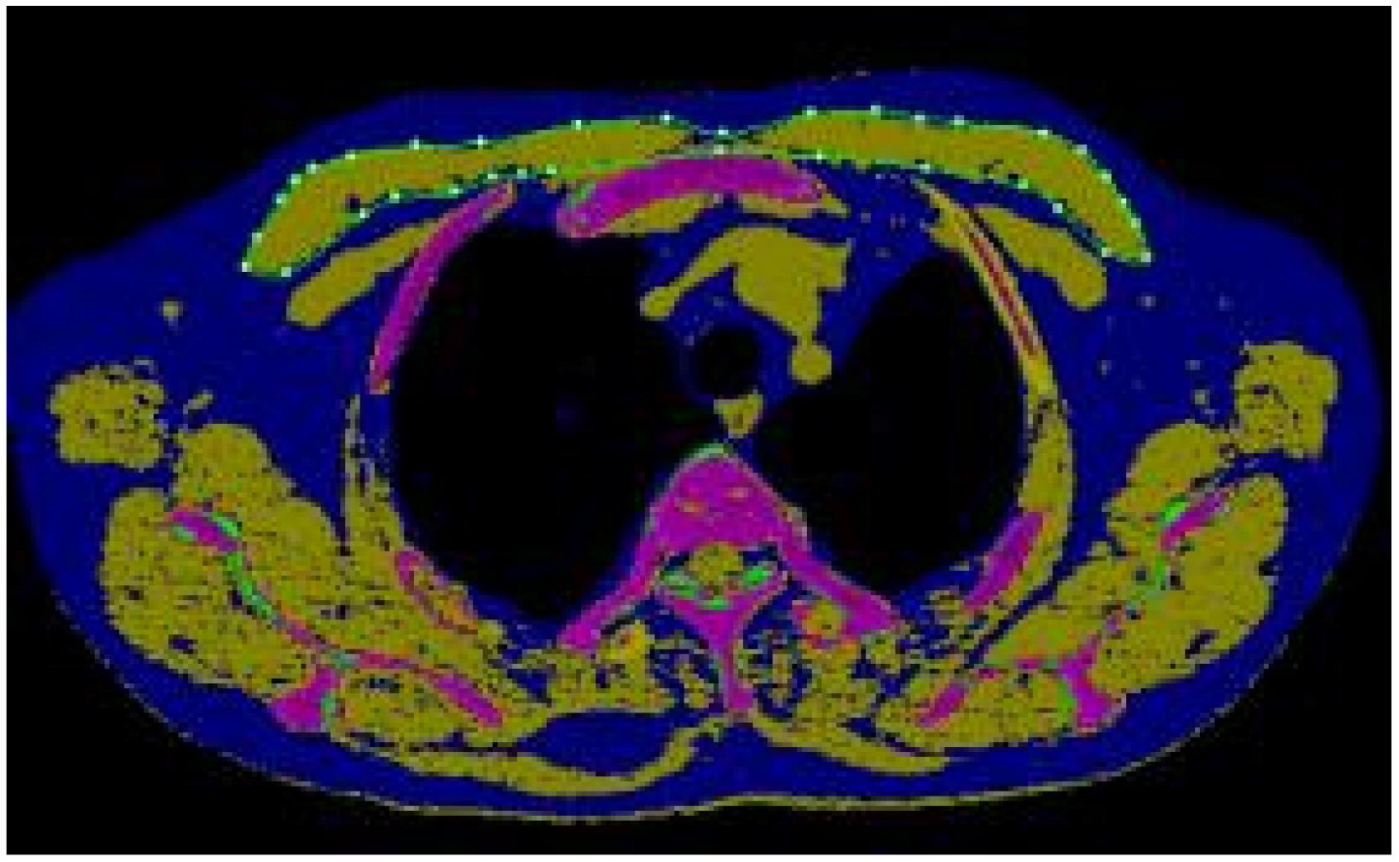
PMcsa measurement diagram.

**Figure 2:**
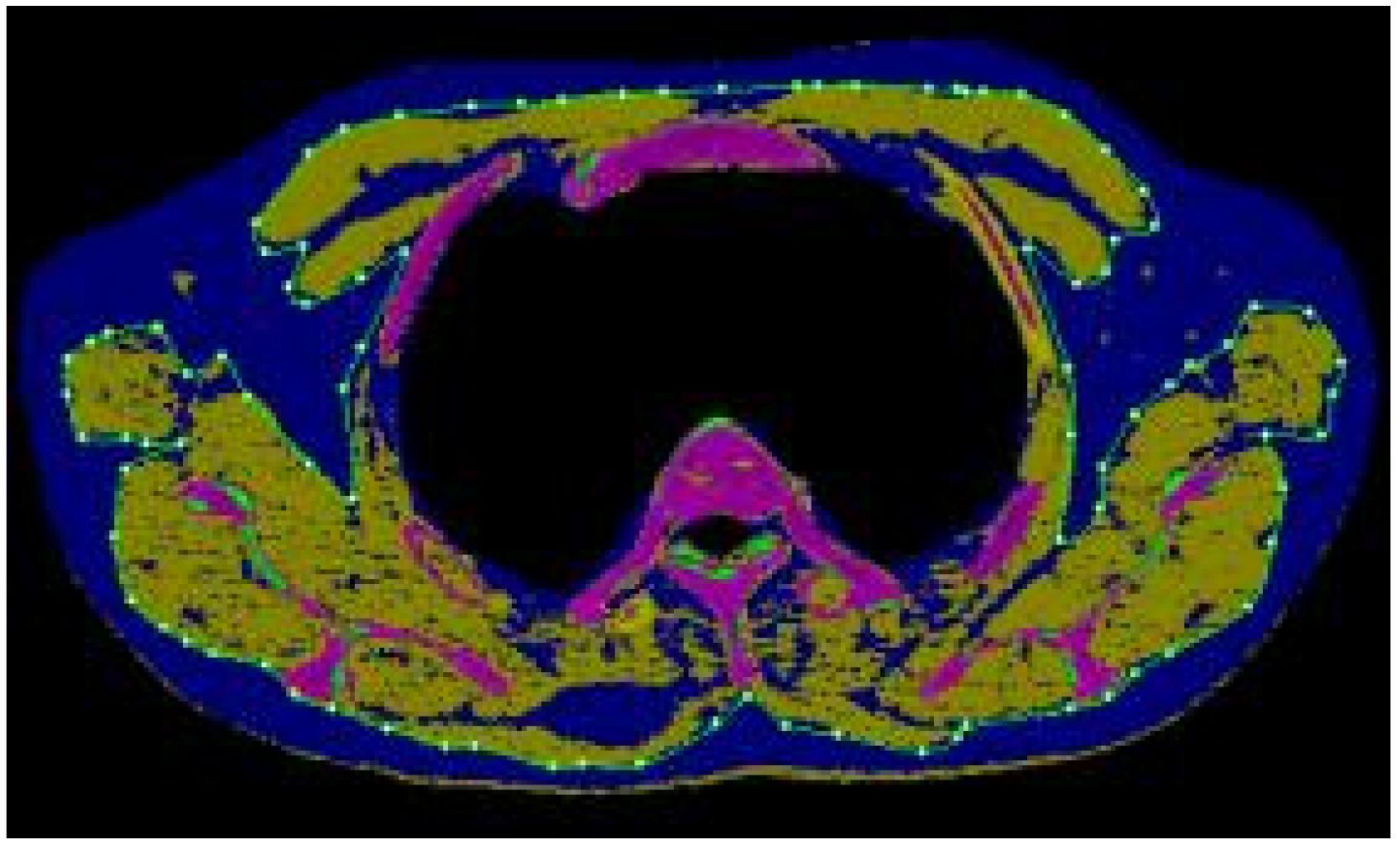
Measurement diagram of subcutaneous fat and total muscle area of all muscle groups in the same layer. The blue area outside the green circle represents subcutaneous fat; the yellow area inside the green circle represents all muscle groups.

**Figure 3:**
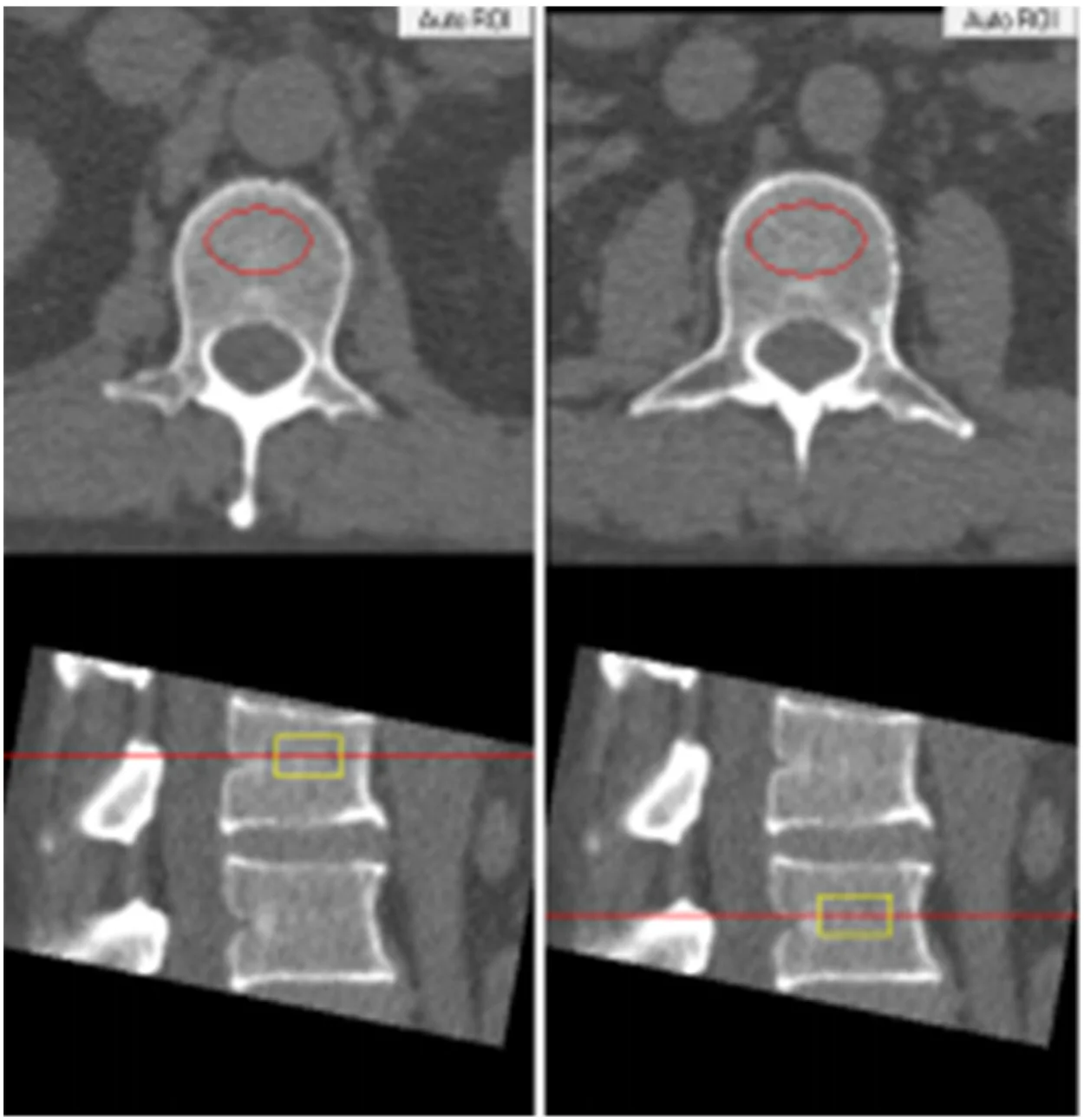
Schematic diagram of bone density measurement.

#### 2.2.3 LAA% measurement

All original chest CT data were imported into the NeuLungCARE-QA software for automated whole-lung segmentation and quantitative analysis. LAA% was defined as the percentage of total lung voxels with attenuation values below −950 HU (LAA−950%), a commonly used density-mask threshold for quantitative CT assessment of emphysema (8). Although the software interface simultaneously displays measurements based on −950 HU and −910 HU (Figure 4), only LAA−950% was used in the analyses reported in this study. For descriptive stratification, participants were categorized according to prespecified LAA−950% cut points as grade 0 (<15%), grade 1 (15% to <25%), grade 2 (25% to <35%), and grade 3 (≥35%). The 15% and 25% cut points were selected with reference to previous quantitative CT studies stratifying emphysema burden, while a threshold of 35% has been used to identify individuals with a high emphysema burden (9, 10). These categories were used as study-specific CT-based strata rather than as a universally established clinical staging system.

**Figure 4:**
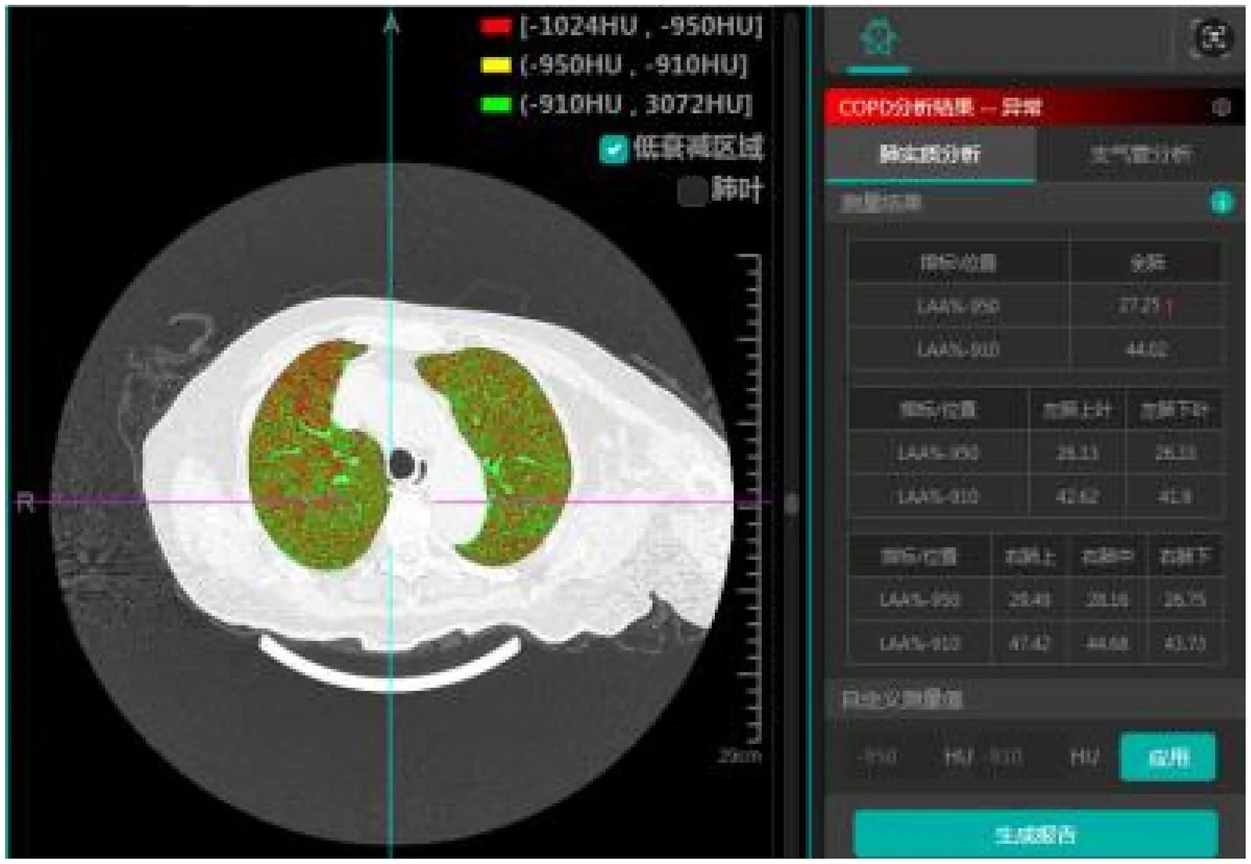
Representative LAA% measurement using NeuLungCARE-QA. The software displays measurements based on both −950 HU and −910 HU; LAA−950% was used for the analyses in the present study.

### 2.3 Quality control

To ensure the scientific rigor and reliability of the results of this study, quality control is implemented throughout the entire process of data collection, processing, and analysis. Research subjects are rigorously screened based on predefined inclusion and exclusion criteria. All chest CT images are acquired using standardized equipment and scanning parameters, and measurements are performed using validated quantitative analysis software. To minimize measurement bias, the manually delineated CT-derived body composition parameters were independently measured by two experienced radiologists who were blinded to the participants' clinical information and LAA%-based group classification. NeuLungCARE-QA was used for automated whole-lung segmentation and LAA% calculation, and the resulting segmentation was reviewed for technical adequacy before analysis. Any substantial discrepancies between the two radiologists were resolved by consensus; when consensus could not be reached, a third senior radiologist was consulted for arbitration. Interobserver agreement for the manually measured CT-derived body composition parameters was assessed using the intraclass correlation coefficient (ICC) based on the initial independent measurements obtained by the two radiologists before consensus. A two-way random-effects model with absolute agreement and single measurements was used, and ICCs with 95% confidence intervals (CIs) were reported. PMI was not assessed separately for interobserver agreement because it was mathematically derived from PMcsa and height. LAA% was not included in the interobserver agreement analysis because it was generated automatically by NeuLungCARE-QA after whole-lung segmentation. All collected clinical data are strictly verified and entered according to relevant diagnostic criteria to minimize the potential impact of information bias on the observed data.

### 2.4 Statistical methods

Data analysis was performed using SPSS version 27.0. Continuous variables with a normal distribution are presented as mean ± standard deviation (SD). Comparisons between two groups were performed using the independent-samples t-test, and comparisons among multiple groups were performed using one-way analysis of variance. Continuous variables with a non-normal distribution are presented as median and interquartile range [M (P25, P75)]. Comparisons between two groups were performed using the Mann–Whitney U test, and comparisons among multiple groups were performed using the Kruskal–Wallis test. Categorical variables are presented as number (percentage), and comparisons among groups were performed using the *χ*^2^ test or Fisher's exact test, as appropriate. For variables showing a significant overall difference among the four LAA% groups, *post hoc* pairwise comparisons were subsequently performed with Bonferroni adjustment for multiple comparisons.

Spearman correlation analysis was used to assess unadjusted associations between quantitative CT (QCT)-derived body composition parameters and LAA%. Covariate-adjusted associations were subsequently evaluated using Gamma generalized linear models with a log link. The Gamma modeling approach was applied because LAA% values included in the regression analyses were strictly positive and showed a right-skewed distribution. Each QCT-derived body composition parameter was evaluated in a separate model with age, sex, and body mass index (BMI) included as covariates. Because the pectoralis major muscle index (PMI) is calculated as the pectoralis major muscle cross-sectional area (PMcsa) divided by height squared, PMcsa and PMI were evaluated in separate models and were not entered simultaneously into the same adjusted model, thereby avoiding collinearity arising from their mathematical relationship. Regression coefficients (β), standard errors (SE), Wald *χ*^2^ statistics, 95% confidence intervals (CIs), and *P* values were reported for the adjusted models.

The discriminative performance of QCT-derived body composition parameters for LAA% grade 3 was evaluated using receiver operating characteristic (ROC) curve analysis. The area under the curve (AUC), 95% CI, optimal cutoff value, sensitivity, and specificity were reported. Internal validation of the ROC analyses was performed using 1,000 bootstrap resamples, from which bootstrap-corrected AUCs and corresponding 95% CIs were obtained. Because the total muscle area of the same-layer muscle group yielded the numerically highest apparent AUC, exploratory DeLong tests were used to compare its AUC with those of the other body composition parameters. Interobserver agreement for manually measured CT-derived body composition parameters was evaluated using intraclass correlation coefficients (ICCs) with 95% CIs. Bootstrap validation and DeLong comparisons were performed using R version 4.3.2, whereas the remaining statistical analyses were performed using SPSS version 27.0. All statistical tests were two-sided, and *P* < 0.05 was considered statistically significant.

## 3 Results

### 3.1 Comparison of general data

The 260 participants were categorized according to CT-derived LAA% into grade 0 (*n* = 114), grade 1 (*n* = 57), grade 2 (*n* = 20), and grade 3 (*n* = 69). No statistically significant differences were observed among the four groups in sex, age, height, weight, or BMI (all *P* > 0.05), as shown in Table 1.

**Table 1:** General characteristics of participants according to LAA% grade.

Characteristic	LAA% grade 0 (*n* = 114)	LAA% grade 1 (*n* = 57)	LAA% grade 2 (*n* = 20)	LAA% grade 3 (*n* = 69)	*χ*^2^/H/F	*P*
Sex					7.486	0.058
Male	88 (77.19)	53 (92.98)	18 (90.00)	56 (81.16)		
Female	26 (22.81)	4 (7.02)	2 (10.00)	13 (18.84)		
Age, years	60 (56, 64)	61 (59, 71)	63 (60, 67)	60 (55, 65)	7.616	0.055
Height, cm	169.54 ± 6.46	172.02 ± 5.28	172.40 ± 8.28	170.64 ± 6.27	2.526	0.058
Weight, kg	71.84 ± 11.01	73.35 ± 8.72	74.30 ± 11.42	71.90 ± 10.88	0.520	0.669
BMI, kg/m^2^	24.91 ± 3.02	24.76 ± 2.47	24.88 ± 2.40	24.59 ± 2.72	0.196	0.899

Normally distributed continuous variables are presented as mean ± SD and were compared using one-way analysis of variance. Non-normally distributed continuous variables are presented as median and interquartile range [M (P25, P75)] and were compared using the Kruskal–Wallis test. Categorical variables are presented as *n* (%) and were compared using the χ^2^ test. BMI, body mass index.

### 3.2 Body composition comparison

Significant between-group differences were observed in PMcsa, PMD, PMI, overall pectoralis major fat infiltration rate, subcutaneous fat content, total muscle area of the same-layer muscle group, and vBMD (all *P* < 0.05). The most pronounced differences were generally observed in participants with LAA% grade 3, who had the lowest PMcsa, PMD, PMI, subcutaneous fat content, total muscle area, and vBMD and the highest pectoralis major fat infiltration rate. However, not all parameters showed a strictly monotonic change across grades 0–3; for example, PMcsa was slightly higher in grade 1 than in grade 0. Therefore, the categorical comparisons should primarily be interpreted as showing more marked body composition abnormalities in the high-LAA% group rather than a uniform dose-response relationship across all grades (Table 2).

**Table 2:** Comparison of QCT-derived body composition parameters according to LAA% grade.

Parameter	LAA% grade 0 (*n* = 114)	LAA% grade 1 (*n* = 57)	LAA% grade 2 (*n* = 20)	LAA% grade 3 (*n* = 69)	H/F	*P*
PMcsa, cm^2^	26.88 (22.98, 32.81)	27.31 (23.79, 31.08)	25.47 (21.92, 29.36)	20.02 (17.06, 21.31)^a^^,^^b^^,^^c^	78.127	<0.001
PMD, HU	24.63 (18.37, 30.14)	25.04 (17.19, 29.35)	22.94 (17.56, 28.23)	18.57 (16.20, 23.92)^a^^,^^b^^,^^c^	18.879	<0.001
PMI, cm^2^/m^2^	9.13 (7.77, 11.19)	9.14 (8.01, 10.41)	8.56 (7.54, 9.72)	6.71 (6.05, 7.52)^a^^,^^b^^,^^c^	82.563	<0.001
Overall pectoralis major fat infiltration rate, %	45.91 (35.68, 57.22)	45.84 (36.89, 59.38)	48.72 (39.16, 57.93)	55.81 (46.72, 65.08)^a^^,^^b^	18.038	<0.001
Subcutaneous fat content, cm^2^	106.67 ± 10.55	103.48 ± 11.25	100.37 ± 12.22^a^	95.41 ± 10.70^a^^,^^b^	15.511	<0.001
Total muscle area of the same-layer muscle group, cm^2^	150.28 ± 12.57	144.51 ± 10.24^a^	135.36 ± 10.78^a^^,^^b^	128.20 ± 8.77^a^^,^^b^^,^^c^	59.904	<0.001
vBMD, mg/cm^3^	104.49 ± 33.10	100.95 ± 23.97	96.38 ± 27.66	91.84 ± 20.56^a^	3.037	0.030

PMcsa, PMD, PMI, and overall pectoralis major fat infiltration rate are presented as median and interquartile range [M (P25, P75)] and were compared using the Kruskal–Wallis test. Subcutaneous fat content, total muscle area, and vBMD are presented as mean ± SD and were compared using one-way analysis of variance.

a Bonferroni-adjusted *P* < 0.05 vs. LAA% grade 0.

b Bonferroni-adjusted *P* < 0.05 vs. LAA% grade 1.

c Bonferroni-adjusted *P* < 0.05 vs. LAA% grade 2.

PMcsa, pectoralis major muscle cross-sectional area; PMD, pectoralis major muscle density; PMI, pectoralis major muscle index; vBMD, volumetric bone mineral density.

### 3.3 Analysis of the correlation between body composition and LAA%

Since LAA% is a continuous variable with a non-normal distribution, Spearman correlation analysis was used. The results showed that LAA% was significantly negatively correlated with PMcsa, PMD, PMI, subcutaneous fat content, total muscle area of the same layer muscle group, and vBMD (r = −0.482, −0.470, −0.502, −0.513, −0.447, −0.441, *P* < 0.05), and significantly positively correlated with the overall fat infiltration rate of the pectoralis major muscle (r = 0.465, *P* < 0.05), see Figure 5.

**Figure 5:**
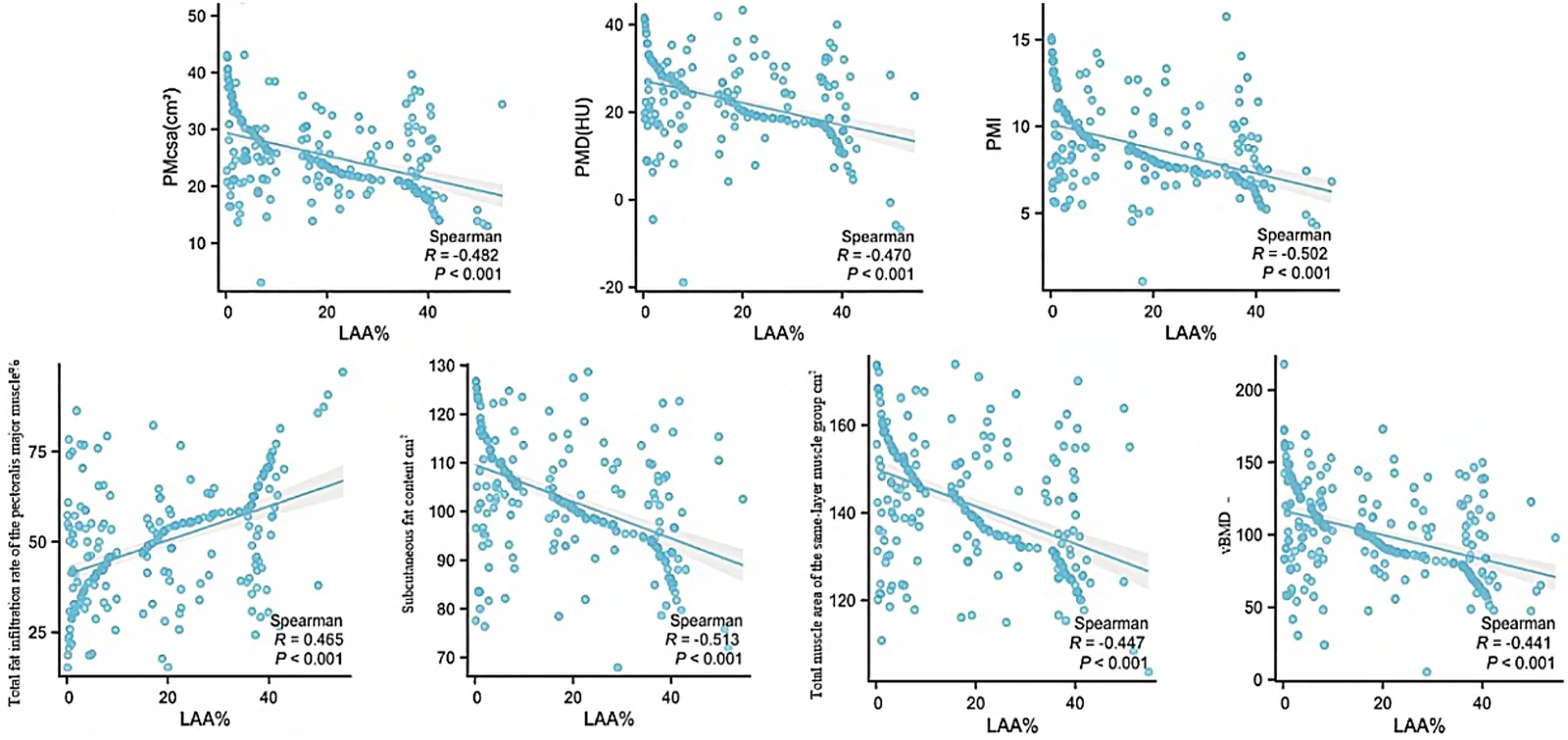
Correlation analysis of body composition with LAA%.

### 3.4 Covariate-adjusted associations between body composition parameters and LAA%

With LAA% as the dependent variable, separate Gamma generalized linear models were constructed for each QCT-derived body composition parameter, with adjustment for age, sex, and BMI. PMcsa and PMI were evaluated in separate models because PMI is mathematically derived from PMcsa and height squared. After adjustment, PMcsa, PMD, PMI, subcutaneous fat content, total muscle area of the same-layer muscle group, and vBMD remained negatively associated with LAA%, whereas the overall pectoralis major fat infiltration rate remained positively associated with LAA% (all *P* < 0.05), as shown in Table 3.

**Table 3:** Associations between QCT-derived body composition parameters and LAA% after adjustment for age, sex, and BMI.

Indicator	Adjusted *β*	SE	Wald χ^2^	95% CI	*P*
PMcsa	−0.106	0.046	5.310	−0.196 to −0.016	0.021
PMD	−0.141	0.061	5.343	−0.261 to −0.021	0.021
PMI	−0.552	0.218	6.412	−0.979 to −0.125	0.011
Overall pectoralis major fat infiltration rate	0.311	0.041	57.538	0.231 to 0.391	<0.001
Subcutaneous fat content	−0.294	0.045	42.684	−0.382 to −0.206	<0.001
Total muscle area of the same-layer muscle group	−0.169	0.040	17.851	−0.247 to −0.091	<0.001
vBMD	−0.033	0.015	4.840	−0.062 to −0.004	0.028

### 3.5 Discriminative performance of body composition parameters for LAA% grade 3

ROC curve analysis showed that the apparent AUCs of the QCT-derived body composition parameters for discriminating LAA% grade 3 ranged from 0.733 to 0.887, indicating moderate-to-good discriminative performance. After 1,000 bootstrap resamples, the bootstrap-corrected AUCs ranged from 0.726 to 0.881, with only small reductions from the apparent AUCs, suggesting limited optimism in the AUC estimates. The total muscle area of the same-layer muscle group had the numerically highest apparent AUC of 0.887 (95% CI: 0.845–0.929) and a bootstrap-corrected AUC of 0.881 (95% CI: 0.838–0.923). Its optimal cutoff value was 138.87 cm^2^, with a sensitivity of 0.899 and a specificity of 0.738. Exploratory DeLong comparisons showed that its AUC was not significantly different from those of PMI (*P* = 0.428), PMcsa (*P* = 0.301), or overall pectoralis major fat infiltration rate (*P* = 0.216), whereas it was significantly higher than those of PMD (*P* = 0.041), subcutaneous fat content (*P* < 0.001), and vBMD (*P* = 0.007). Therefore, although total muscle area showed the highest numerical AUC, it should not be considered statistically superior to all other body composition parameters. Detailed ROC results and bootstrap validation are shown in Table 4 and Figure 6.

**Table 4:** Discriminative performance and bootstrap internal validation of QCT-derived body composition parameters for LAA% grade 3.

Indicator	Apparent AUC	95% CI	Bootstrap-corrected AUC	Bootstrap 95% CI	Cutoff value	Sensitivity	Specificity	DeLong P*
PMcsa	0.857	0.805–0.909	0.851	0.799–0.902	23.01 cm^2^	0.884	0.764	0.301
PMD	0.815	0.748–0.882	0.808	0.741–0.873	17.40 HU	0.725	0.901	0.041
PMI	0.864	0.813–0.916	0.858	0.807–0.908	8.05 cm^2^/m^2^	0.870	0.733	0.428
Overall pectoralis major fat infiltration rate	0.843	0.776–0.909	0.837	0.771–0.901	58.74%	0.797	0.927	0.216
Subcutaneous fat content	0.733	0.666–0.801	0.726	0.658–0.793	95.65 cm^2^	0.826	0.555	<0.001
Total muscle area of the same-layer muscle group	0.887	0.845–0.929	0.881	0.838–0.923	138.87 cm^2^	0.899	0.738	—
vBMD	0.768	0.697–0.839	0.761	0.691–0.829	96.25 mg/cm^3^	0.681	0.845	0.007

Bootstrap-corrected AUCs and 95% CIs were obtained using 1,000 bootstrap resamples. DeLong *P* values represent exploratory pairwise comparisons between each parameter and the total muscle area of the same-layer muscle group, which had the numerically highest apparent AUC.

AUC, area under the curve; CI, confidence interval; PMcsa, pectoralis major muscle cross-sectional area; PMD, pectoralis major muscle density; PMI, pectoralis major muscle index; vBMD, volumetric bone mineral density.

* DeLong P values represent exploratory pairwise comparisons between each parameter and the total muscle area of the same-layer muscle group, which had the numerically highest apparent AUC.

**Figure 6:**
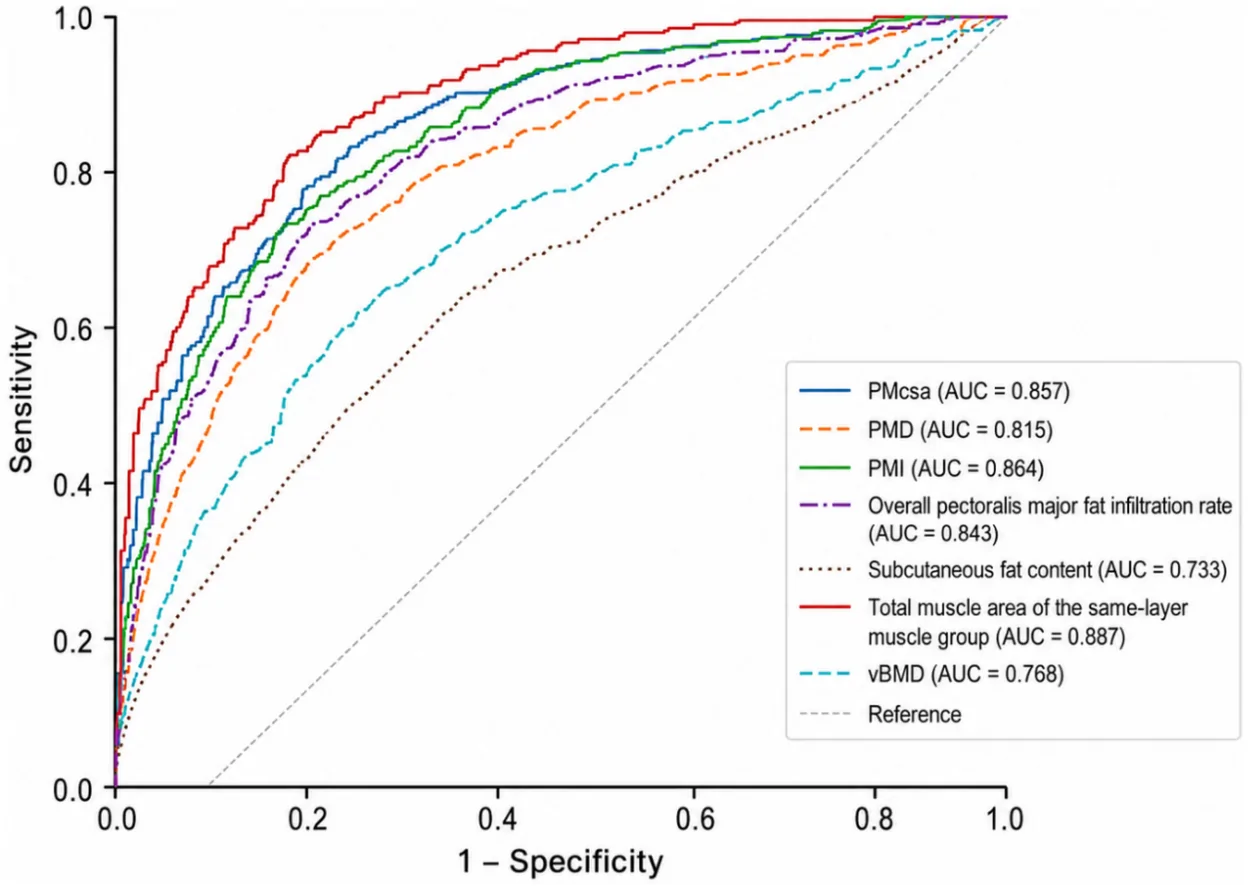
ROC curves of QCT-derived body composition parameters for discriminating LAA% grade 3. Bootstrap-corrected AUC estimates are reported in Table 4.

### 3.6 Interobserver agreement

Interobserver agreement between the two radiologists was high for all manually measured CT-derived body composition parameters. The ICC was 0.956 (95% CI: 0.944–0.966) for PMcsa, 0.932 (95% CI: 0.914–0.946) for PMD, 0.921 (95% CI: 0.900–0.938) for overall pectoralis major fat infiltration rate, 0.948 (95% CI: 0.934–0.959) for subcutaneous fat content, 0.969 (95% CI: 0.961–0.976) for total muscle area of the same-layer muscle group, and 0.974 (95% CI: 0.967–0.980) for vBMD. PMI was not evaluated separately because it was mathematically derived from PMcsa and height. LAA% was not included in the interobserver agreement analysis because it was generated automatically by NeuLungCARE-QA after whole-lung segmentation.

## 4 Discussion

CT-derived LAA% provides an objective quantitative measure of low-attenuation lung parenchyma and is widely used to characterize emphysema-related structural burden (8). In parallel, routine chest CT can also provide quantitative information on skeletal muscle, adipose tissue, and bone-related parameters (11, 12). Because the present cohort was derived from health examination records and smoking history and pulmonary function data were not consistently available, the findings should be interpreted primarily within a CT imaging framework rather than as evidence regarding clinically diagnosed COPD. In the present study, multiple QCT-derived body composition parameters were significantly associated with LAA%, and several parameters demonstrated good ability to discriminate participants with LAA% grade 3. These findings suggest that simultaneous assessment of pulmonary attenuation and body composition from the same CT examination may provide complementary information regarding pulmonary structural burden and systemic body composition.

PMcsa is a direct measurement index reflecting local muscle quantity, and measurements obtained at the level of the upper margin of the aortic arch have shown good correlation with whole-body fat-free soft tissue mass. Previous studies in COPD populations have also suggested that PMcsa may serve as a useful CT-derived surrogate for skeletal muscle assessment (12). PMI eliminates the influence of individual body size differences on muscle absolute area through height correction, making muscle reserve comparisons between different body types more comparable (13). The decline in PMD essentially reflects the degree of intramuscular fat infiltration and fibrosis, serving as a core indicator for assessing muscle quality (14). Meanwhile, the total muscle area of the same-layer muscle group breaks through the limitations of single muscle assessment, enabling a more comprehensive reflection of the body's overall muscle reserve level (12). The above four parameters reflect the quantity, quality, and overall reserve status of the body's skeletal muscle from different dimensions, jointly constituting the core indicator system for QCT assessment of muscle health status. The between-group findings should not be interpreted as demonstrating a uniform dose-response relationship across the four LAA% grades. Although participants in LAA% grade 3 generally showed the most pronounced abnormalities in skeletal muscle measures, changes across the lower LAA% grades were not strictly monotonic. For example, median PMcsa was 26.88 cm^2^ in grade 0 and 27.31 cm^2^ in grade 1 before decreasing to 25.47 cm^2^ in grade 2 and 20.02 cm^2^ in grade 3. Similar non-monotonic patterns across the lower grades were observed for PMD, PMI, and pectoralis major fat infiltration. In contrast, subcutaneous fat content, total muscle area, and vBMD showed more consistently decreasing values across increasing LAA% grades. Taken together, these findings suggest that the categorical group differences were driven largely by the more pronounced body composition abnormalities observed in LAA% grade 3 rather than by a uniform stepwise deterioration across all grades. Nevertheless, the significant Spearman correlations and covariate-adjusted associations with continuous LAA% indicate that body composition parameters remained associated with overall CT-defined low-attenuation burden. These findings are broadly consistent with previous reports describing skeletal muscle abnormalities in COPD populations (15). Accordingly, the present muscle-related findings should primarily be interpreted as extending these established observations within a multidimensional QCT-based assessment rather than as identifying a previously unrecognized association. Skeletal muscle contributes to systemic metabolic and redox homeostasis. Previous studies in COPD populations have linked skeletal muscle dysfunction with oxidative stress and systemic inflammation, which may contribute to muscle protein catabolism and reduced muscle quality (15, 16). These mechanisms provide a plausible biological context for the association between lower muscle-related CT measures and greater LAA% observed in the present study. However, because the present analysis was cross-sectional, the direction and causality of this relationship cannot be established.

Meanwhile, individuals with severe emphysema continuously release inflammatory factors such as tumor necrosis factor-α from their lungs, which activate the NF-κB pathway after entering the circulation, promoting the expression of related enzymes, leading to accelerated muscle fiber protein degradation and muscle loss; muscle loss can also further weaken the body's anti-inflammatory reserves, accelerating the progression of emphysema (16).

The present study also showed that the overall pectoralis major fat infiltration rate was higher in participants with LAA% grade 3 (*P* < 0.05). Greater pectoralis major fat infiltration may reflect myosteatosis and altered systemic metabolic status. Previous studies have implicated ectopic lipid accumulation, insulin resistance, and inflammatory signaling in skeletal muscle dysfunction and COPD-related systemic abnormalities (17). These mechanisms may provide a biological context for the positive association between pectoralis major fat infiltration and LAA% observed in the present study, but they should not be interpreted as evidence that intramuscular fat directly causes emphysema progression. Impaired epithelial repair has also been discussed in COPD-related mechanistic studies (18), although this pathway was not directly assessed in the present cohort.

This study also found that subcutaneous fat content was lower with increasing CT-derived LAA% burden (*P* < 0.05). Subcutaneous fat is the main source of adiponectin (19). Adiponectin has anti- inflammatory, anti-apoptotic, and insulin-sensitizing effects (20). Previous studies in COPD populations have reported reduced subcutaneous fat stores and altered adipokine profiles in individuals with greater disease burden, which may provide a potential biological context for the association observed in the present study (20). Meanwhile, subcutaneous fat serves as an important energy reserve that helps the body cope with chronic catabolic stress. When subcutaneous fat stores are markedly reduced, the body may enter a state of negative nitrogen balance, promoting muscle protein breakdown for energy and thereby aggravating muscle loss and potentially contributing to greater emphysema-related structural burden (21). Reduced bone density in individuals with emphysema-related structural changes may be influenced by multiple factors, including age, nutritional status, vitamin D status, systemic inflammation, smoking exposure, and medications affecting bone metabolism (22). Therefore, the observed association between vBMD and LAA% should be interpreted as an imaging-based association rather than evidence of a direct causal relationship.

Both the unadjusted Spearman correlation analyses and the covariate-adjusted Gamma generalized linear models supported significant associations between QCT-derived body composition parameters and LAA%. After adjustment for age, sex, and BMI, lower PMcsa, PMD, PMI, subcutaneous fat content, total muscle area of the same-layer muscle group, and vBMD remained significantly associated with higher LAA%, whereas greater pectoralis major fat infiltration remained positively associated with LAA% (all *P* < 0.05). Each body composition parameter was evaluated in a separate adjusted model, thereby avoiding simultaneous inclusion of mathematically related parameters such as PMcsa and PMI. ROC curve analysis further showed moderate-to-good discriminative performance of the evaluated QCT-derived body composition parameters for LAA% grade 3. Bootstrap internal validation produced corrected AUCs ranging from 0.726 to 0.881, which were close to the apparent AUCs and suggested limited optimism in the original estimates. The total muscle area of the same-layer muscle group had the numerically highest apparent and bootstrap-corrected AUCs (0.887 and 0.881, respectively). However, exploratory DeLong comparisons showed that its AUC did not differ significantly from those of PMI, PMcsa, or pectoralis major fat infiltration rate, although it was significantly higher than those of PMD, subcutaneous fat content, and vBMD. Therefore, the present findings support the complementary discriminative value of several CT-derived body composition measures rather than the statistical superiority of a single parameter. These findings indicate that QCT-derived body composition parameters are associated with CT-defined LAA% burden and may provide complementary imaging information for distinguishing individuals with higher LAA%, particularly those in LAA% grade 3.

This study still has certain limitations. This study is a single-center retrospective design, and the external validity of the results may be limited. Although bootstrap resampling provided internal validation of the ROC estimates, the optimal cutoff values and their corresponding sensitivity and specificity were derived and evaluated within the same dataset; therefore, these estimates may be optimistic and should be interpreted cautiously. The DeLong AUC comparisons should also be regarded as exploratory. External validation in an independent cohort remains necessary before these discriminative findings can be generalized to other populations. Accordingly, the present findings should not be interpreted as establishing clinically actionable biomarkers or novel pathophysiological mechanisms; rather, they provide exploratory imaging evidence that requires further prospective and clinical validation. Smoking history and pulmonary function measurements were not consistently available in the retrospective health examination records. Therefore, smoking exposure could not be adequately adjusted for, and clinical COPD status or airflow limitation could not be systematically established. Accordingly, the present findings should be interpreted as associations between CT-derived body composition and CT-defined LAA% rather than as associations specifically within a clinically diagnosed COPD population. In addition, detailed medication histories that could affect bone metabolism, including thyroid hormone therapy, postmenopausal hormone replacement therapy, systemic corticosteroids, calcium/vitamin D supplementation, and bisphosphonate therapy, were not consistently available in the retrospective records. Therefore, potential confounding by these medications could not be fully controlled, and the association between vBMD and LAA% should be interpreted cautiously. Although age, sex, and BMI were included in the adjusted analyses, residual confounding from unavailable factors, including smoking exposure, pulmonary function, and medications affecting bone metabolism, cannot be completely excluded. The measurement of body composition in this study was based on only a single level at the upper margin of the aortic arch. Although this level has clear anatomical landmarks and high measurement reproducibility, the measurement at a single level may be subject to selection bias. Additionally, this study did not include clinical endpoints such as quality of life and follow-up prognosis, and the causal relationship between changes in body composition and long-term clinical outcomes of emphysema remains unclear.

Future studies could further validate these findings in multicenter prospective cohorts using multi-level or three-dimensional segmentation techniques and incorporating relevant clinical endpoints to investigate longitudinal associations between changes in body composition and subsequent disease outcomes.

In summary, QCT-derived body composition parameters are associated with CT-defined LAA% burden, including differences in muscle quantity and quality, fat distribution, and bone mineral density. Additionally, PMcsa, PMD, PMI, overall pectoralis major fat infiltration rate, subcutaneous fat content, total muscle area of the same-layer muscle groups, and vBMD showed good discriminative performance for LAA% grade 3, suggesting that these CT-derived body composition parameters may provide complementary information for identifying individuals with a greater CT-defined emphysema burden.

## Data Availability

The original contributions presented in the study are included in the article/Supplementary Material, further inquiries can be directed to the corresponding author.
